# Prevalence of *Neospora* spp. in donkeys in China

**DOI:** 10.1051/parasite/2018018

**Published:** 2018-03-20

**Authors:** Wei Cong, Lan-Bi Nie, Si-Yuan Qin, Wei-Li Wang, Ai-Dong Qian, Qing-Feng Meng

**Affiliations:** 1 College of Marine Science, Shandong University at Weihai, Weihai, Shandong Province 264209 PR China; 2 College of Animal Science and Technology, Jilin Agricultural University, Changchun, Jilin Province 130118 PR China; 3 General Station for Surveillance of Wildlife Diseases & Wildlife Borne Diseases, State Forestry Administration (SFA), Shenyang 110034 PR China; 4 Jilin Entry-Exit Inspection and Quarantine Bureau, ChangChun, Jilin Province 130000 PR China

**Keywords:** *Neospora*, cELISA, detection, donkeys, China

## Abstract

This study was conducted to detect specific anti-*Neospora* antibodies using a commercial competitive-inhibition ELISA kit, and to evaluate the risk factors for *Neospora* spp. infection. Out of a total of 2,228 donkey sera collected in three provinces in China, 211 (9.5%) were found to be positive for anti-*Neospora* antibodies. Statistical analysis revealed that age (*p* = 0.019, OR = 1.62, 95%CI: 1.08-2.44), feeding status (*p* < 0.001, OR = 3.79, 95%CI: 2.65-5.43), miscarriage history (*p* = 0.006, OR = 2.56, 95%CI: 1.27-4.01), and contact with dogs (*p* < 0.001, OR = 2.69, 95%CI: 1.86-3.88) were significant risk factors for *Neospora* spp. infection. This is the first evidence of *Neospora* infection in donkeys in China.

## Abstract (fr)

Cette étude a été menée pour détecter les anticorps spécifiques anti-*Neospora* par un kit ELISA compétitif d’inhibition compétitive et évaluer les facteurs de risque des infections par *Neospora* spp. Sur un total de 2228 sérums d’ânes collectés dans trois provinces de Chine, 211 (9,5 %) ont été trouvés positifs pour les anticorps anti-*Neospora*. L’analyse statistique a révélé que l’âge (*p* = 0,019, OR = 1,62, IC à 95 % : 1,08-2,44), l’alimentation (*p* < 0,001, OR = 3,79, IC à 95 % : 2,65-5,43), l’historique d’avortements (*p *= 0,006, OR = 2,56, IC à 95 % : 1,27-4,01), et le contact avec les chiens (*p* < 0,001, OR = 2,69, IC à 95 % : 1,86-3,88) étaient des facteurs de risque significatifs pour l’infection à *Neospora* spp. Ceci est la première preuve d’infection à *Neospora* chez les ânes en Chine.

## Introduction

*Neospora* spp. are globally distributed obligate intracellular parasites [22] and are closely related to *Toxoplasma gondii* and *Sarcocystis* spp., belonging to the phylum Apicomplexa of the Sarcocystidae family [7]. *Neospora* can infect a wide variety of hosts [8]. Dogs, coyotes and dingoes are definitive hosts of *Neospora* spp. [13,14,19], and certain other mammal species, including cattle and other ruminants, canids and horses can serve as intermediate hosts [10].

Equine neosporosis caused by two species of *Neospora* (*Neospora caninum* and *Neospora hughesi*), is accompanied by neurologic symptoms and reproductive loss [9,21]. Donkeys (*Equus asinus*) are closely related to horses and can be infected by some equine pathogens such as the protozoa *Theileria equi* and *Babesia caballi* [18]. Seroprevalences of *Neospora* spp. in donkeys have only been reported from Southern Italy [17], Nigeria [5], Brazil [11] and Mexico [1]. However, until now, no information was available about the prevalence of this protozoal disease in donkeys from China. Thus, the aim of this study was to detect antibodies to *Neospora* ssp. in donkeys from three provinces in China, and to evaluate the risk factors associated with *Neospora* seroprevalence.

## Materials and methods

**Figure 1:**
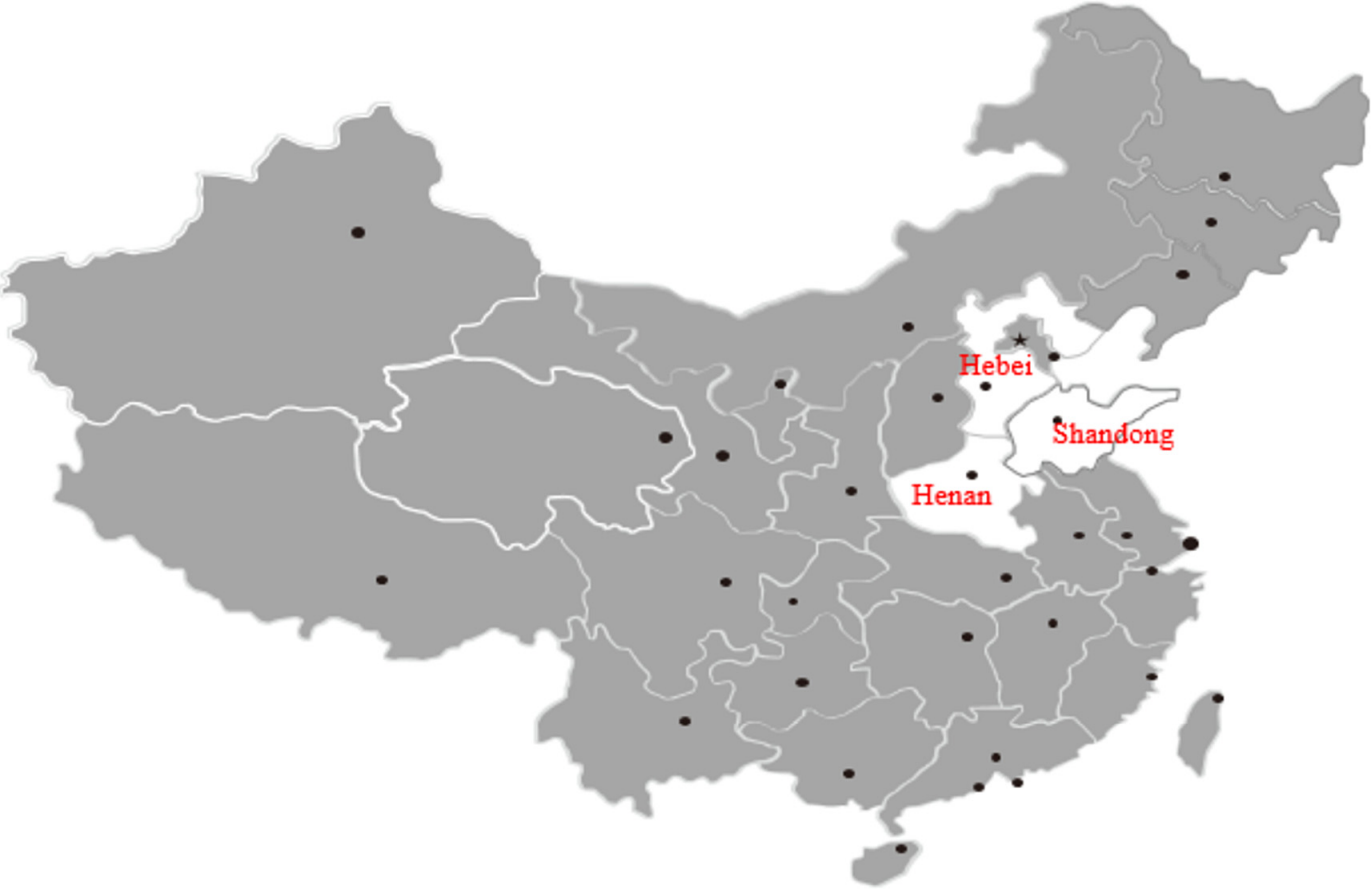
Map of China showing the geographical regions of Hebei, Henan and Shandong provinces where donkeys were sampled.

Serum samples were randomly collected from the jugular vein of 2,228 donkeys from Shandong province (4°23′∼38°24′ N, 114°48′∼122°42′ E), Henan province (31°23′∼36°22′ N, 110°21′∼116°39′ E), and Hebei province (36°05′∼42°40′ N, 113°27′∼119°50′ E) between November 2015 and June 2017 by local veterinary practitioners (Figure 1). Donkeys from each farm were selected randomly using a table of random digits. Several large-scale farms (with more than 300 animals) were not included because the owner did not authorize us to collect samples. Approximately 30% donkeys at each farm were sampled. All of the animals sampled were clinically healthy. Serum samples from backyard donkeys were randomly collected when authorization was obtained from the owners of the donkeys. Serum was obtained through centrifugation at 3000 ×*g* for 5 min and stored at −20 °C until tested. Information about breeds, gender, age, contact with dogs, miscarriage history, and feeding status was acquired from the owners.

A commercial competitive-inhibition enzyme-linked immunosorbent assay kit (cELISA) (VMRD, Pullman, WA, USA) was used to detect *Neospora* antibodies, according to the manufacturer’s instructions [17]. The serum was tested in duplicate and considered positive when the percent inhibition values of both runs were more than 30%.

For the statistical analysis, the SPSS 18.0 software package (IBM, Armonk, NY, USA) was used. The Fisher exact test was used to compare the frequencies among groups. Bivariate and multivariate logistic analyses were used to assess the association between the characteristics of the subjects and the *Neospora* infection. Variables were included in the multivariate logistic analysis if they had a *p* value of equal to or less than 0.35 in the bivariate logistic analysis [1]. A *p* value less than 0.05 was considered statistically significant. Odds-ratios (ORs) with 95% confidence intervals based on likelihood ratio statistics were calculated.

This study was approved by the Animal Ethics Committee of Jilin Agricultural University. Serum samples were collected and handled in accordance with the requirements of the Animal Ethics Procedures and Guidelines of the People’s Republic of China.

## Results and discussion

Commercial competitive-inhibition ELISA kits have been used widely to detect *Neospora* antibodies in many kinds of animals including donkeys [17] due to the method’s high accuracy, sensitivity and accessibility [4].We therefore first used this method to assess the serologic frequency of antibodies to *Neospora* in donkeys from China. Out of a total of 2,228 donkeys, 211 (9.5%) were found to be positive for *Neospora* antibodies. General data for the 2,228 donkeys studied and seroprevalence of *Neospora* spp. infection are shown in Table 1.

**Table 1:** General data for the 2,228 donkeys studied and seroprevalence of *Neospora spp.* infection.

Variable	Category	No. tested	No. positive	Prevalence (%)	*p*-value
Breed	Dezhou	1125	111	9.9	0.383
	Wutou	756	63	8.3	
	Sanfen	347	37	10.7	
Age	≤1 year	466	57	12.2	0.073
	1-5 year	1047	92	8.8	
	≥5 year	715	62	8.7	
Gender	Male	1181	104	8.8	0.255
	Female	1047	107	10.2	
Province	Shandong	847	84	9.9	0.824
	Hebei	626	59	9.4	
	Henan	755	68	9.0	
Feeding status	Backyard	235	68	28.9	< 0.001
	Farm	1993	143	7.2	
Sampling year	2015	615	59	9.6	0.919
	2016	1027	99	9.6	
	2017	586	53	9.0	
Season	Spring	304	24	7.9	0.578
	Summer	639	61	9.5	
	Autumn	724	66	9.1	
	Winter	561	60	10.7	
Miscarriage history	Yes	112	20	17.9	0.002
	No	2116	191	9.0	
Contact with dogs	Yes	246	62	25.2	< 0.001
	No	1982	149	7.5	
Total		2228	211	9.5	

In the present study, age of donkeys was a significant risk factor for this parasitic infection, on the basis of multivariate logistic analysis. The donkeys in age categories ≤1 year (*p* = 0.019, OR = 1.62, 95%CI: 1.08-2.44) were found to have a significantly higher seroprevalence than the other age groups, suggesting that donkeys were exposed to the parasite infection at the early stages of their lives. The same phenomenon was found in a previous study [17]. Moreover, maternal antibodies may have been ingested by colostrum, which may give positive results. This is because the variable is < 1 year, which could include a few months of life and thereby influence the result. Interestingly, nine female donkeys and their offspring were found to be positive for *Neospora* antibodies. This phenomenon leads us to consider possible vertical transmission of *Neospora* infection in donkeys in further studies.

*Neospora* infection can induce clinical neosporosis disease, which notably presents as abortion in ruminants. Worldwide, these abortions are the main reason for economic loss to both the dairy and beef industries [6,15]. In the present study, the result of multivariate logistic analysis showed that donkeys with a history of miscarriages have a significantly higher *Neospora* seroprevalence than those without (*p* = 0.006, OR = 2.56, 95%CI: 1.27-4.01). Although no direct evidence showed that the miscarriages of donkeys were caused by *Neospora* infection, prevention and control of *Neospora* infection should be carried out in the process of raising donkeys. Moreover, further studies should be conducted to explore the association between miscarriage and *Neospora* infection.

Dogs, the definitive hosts of *Neospora*, play an important role in the transmission of *N. caninum*, discharging oocysts into the environment [16,20,22], which is a main risk factor for the occurrence of miscarriages and stillbirths associated with *N. caninum* in ruminants and other intermediate hosts [2,3,12]. Not surprisingly, the donkeys have a significantly higher seroprevalence when they have contact with dogs compared to those without contact (*p* < 0.001, OR = 2.69, 95%CI: 1.86-3.88). In China, dogs often act as guard animals on farms, so this may increase the possibility of *N. caninum* infection. Thus, more protective measures should be implemented to reduce *N. caninum* infection in donkeys, such driving stray dogs from the farms, etc.

In the present study, backyard donkeys had a significantly higher seroprevalence than those feeding on farms (*p* < 0.001, OR = 3.79, 95%CI: 2.65-5.43). The most likely explanation is that donkeys feeding in backyards have greater contact with dogs because many dogs are raised in the owner’s home as guard animals. However, all farms were positive for the presence of *Neospora* antibodies. It is therefore impossible to evaluate the role of farms as a risk factor. Further studies should be conducted to explore the role played by neosporosis in reproductive and economic losses to donkey breeding in these regions.

## Conclusions

This is the first report of *Neospora* seroprevalence and risk factors associated with *Neospora* infection in donkeys from China, providing baseline data for designing and evaluating prevention and control measures. More studies are needed to understand and evaluate the occurrence of transplacental transmission throughout pregnancy. Similarly, the occurrence of neurological diseases or fetal loss in the life of congenitally infected donkeys should also be investigated.

## Conflict of interest

The authors declare that they have no conflicts of interest in relation to this article.
